# HER2-Targeted Multifunctional Silica Nanoparticles Specifically Enhance the Radiosensitivity of HER2-Overexpressing Breast Cancer Cells

**DOI:** 10.3390/ijms19030908

**Published:** 2018-03-19

**Authors:** Haruka Yamaguchi, Kazuhide Hayama, Ichiro Sasagawa, Yasuo Okada, Tomoyuki Kawase, Norio Tsubokawa, Makoto Tsuchimochi

**Affiliations:** 1 Department of Life Science Dentistry, The Nippon Dental University, 1-8 Hamauracho, Chuoku, Niigata 951-8580, Japan; harukay@ngt.ndu.ac.jp; 2 Department of Oral and Maxillofacial Radiology, The Nippon Dental University School of Life Dentistry at Niigata, Niigata 951-8580, Japan; hayama@ngt.ndu.ac.jp; 3 Advanced Research Center, The Nippon Dental University School of Life Dentistry at Niigata, Niigata 951-8580, Japan; ichsasgw@ngt.ndu.ac.jp; 4 Department of Pathology, The Nippon Dental University School of Life Dentistry at Niigata, Niigata 951-8580, Japan; yokada@ngt.ndu.ac.jp; 5 Division of Oral Bioengineering, Department of Tissue Regeneration and Reconstitution, Niigata University Graduate School of Medical and Dental Sciences, Niigata 951-8541, Japan; kawase@dent.niigata-u.ac.jp; 6 Faculty of Engineering, Niigata University, Niigata 950-2181, Japan; ntsuboka@eng.niigata-u.ac.jp; 7 Emeritus Professor, The Nippon Dental University, Tokyo 102-8159, Japan

**Keywords:** silica nanoparticle, HER2, radiosensitizer, SK-BR3

## Abstract

We investigated the effects of targeted functionalized silica nanoparticles on the radiosensitivity of cancer cells. Better control of the local concentration of silica nanoparticles may facilitate their use as an adjuvant in conjunction with ionizing radiation to target cancer cells while preventing damage to normal cells. Hyperbranched polyamidoamine (PAMAM) was grafted onto the surface of amorphous silica nanoparticles to functionalize them. The PAMAM-coated silica nanoparticles (PCSNs) were then conjugated with fluorescent dyes. Anti-HER2 antibodies were covalently attached to the labeled PCSNs. The HER2-overexpressing SK-BR3 breast cancer cell line was incubated in medium containing the PCSN probes. After incubation; the cells were exposed to X-ray radiation. Cells were counted in all samples using cell proliferation assays; and apoptotic cells were detected. The cell survival results showed that the combination of the targeted PCSN probes and radiation reduced the survival rate of SK-BR3 cells to a greater extent than when either PCSN probes, PCSNs or radiation were applied individually. The results also showed an increase in apoptosis in the SK-BR3 cells that internalized the PCSN probes and were then irradiated. Based on these data, PCSN probes act as specific radiosensitizing agents for HER2-overexpressing cells.

## 1. Introduction

Radiotherapy is an indispensable treatment for patients with cancer. Nearly half of cancer patients undergo radiotherapy to cure their disease [1,2]. Despite the benefits of radiotherapy in enhancing outcomes and reducing the mortality rate, many patients suffer from unfavorable side effects [2,3,4]. These side effects reduce the efficiency of radiotherapy due to incomplete planned dose distribution and result in lower baseline quality of life [2,3,4]. The use of radiosensitizers is one solution to maximize the efficiency of radiotherapy. Several drugs such as 5-fluorouracil (5-FU), cisplatin and gemcitabine have been used to enhance the effects of radiotherapy [5]. Although a new clinical molecular-targeted strategy—epidermal growth factor receptor (EGFR) combined with HER2 [5]—has been introduced, it is necessary to identify more effective radiosensitizers to increase the efficiency of radiotherapy and to reduce radiation damage to surrounding normal tissues.

Recently, nanoparticles have been widely studied. Gold nanoparticles, polysilsesquioxane, upconverting nanoparticles, and silica nanoparticles have been used for imaging, transport, drug delivery, and as radiosensitizers [6,7,8]. In particular, gold nanoparticles have emerged as promising radiosensitizers that provide significant radiation dose enhancement [9]. Densely packed metal particles selectively scatter and/or absorb high-energy gamma/X-ray radiation [3].

Among the numerous types of nanoparticle probes that have been developed, silica nanoparticles have been utilized and tested as novel tools to deliver therapeutic agents into targeted organs or cells due to their excellent biocompatibility, optical transparency, easily controlled size and facile surface functionalization [10,11,12]. Furthermore, diagnostic probes composed of silica nanoparticles have already been approved for phase 1 human clinical trials, and the first clinical trial investigating modified fluorescent core-shell silica nanoparticles is in progress [13,14].

It is well known that the inhalation of crystalline silica nanoparticles can cause silicosis, bronchitis or cancer, and high concentrations of silica nanoparticles are toxic [12,13], which are important factors that should be considered before use. However, although crystalline and amorphous silica have the same molecular formula (SiO_2_), their structures differ, and they exhibit different biological behaviors. Amorphous silica nanomaterials are generally thought to be safe and nontoxic [15,16]. It is reported that internalized mesoporous silica nanoparticles can release reactive oxygen species [17]. If functionalized amorphous silica nanomaterials are suitable for use as radiosensitizers, they can be used for theranostic strategies, as our previous study showed that polyamidoamine (PAMAM)-coated silica nanoparticles (PCSNs) might function as dual imaging probes [18,19]. In our preliminary data, we found that PCSN probes were internalized by targeted HER2-overexpressing breast cancer cells, which implies that better control of the local concentration of silica nanoparticles may facilitate their use as an adjuvant in conjunction with ionizing radiation to target cancer cells while avoiding damage to normal cells. Therefore, we investigated the effectiveness of targeted PCSNs for cancer cell radio sensitization.

## 2. Results

### 2.1. PCSN Probes

**Figure 1:**
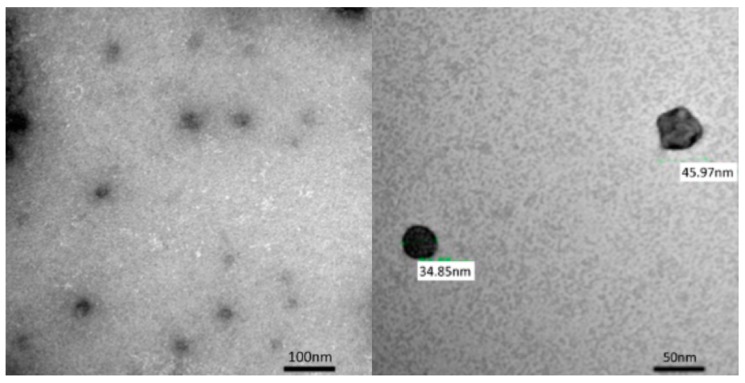
The size of the PAMAM-coated silica nanoparticles, PCSNs prepared was evaluated using a transmission electron microscope (TEM). The PCSN size was approximately 40 nm (between 35 nm and 46 nm).

**Figure 2:**
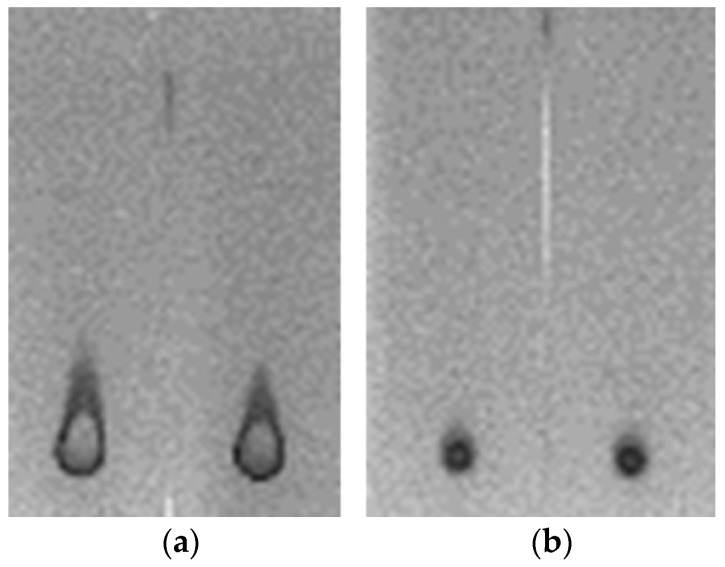
(**a**) Alexa 488 fluorescent dye was run on thin-layer chromatography (TLC) plates, each 2.5 cm × 7.5 cm; (**b**) Conversely, the Alexa 488 of PCSN probes remained at the initial spot.

**Figure 3:**
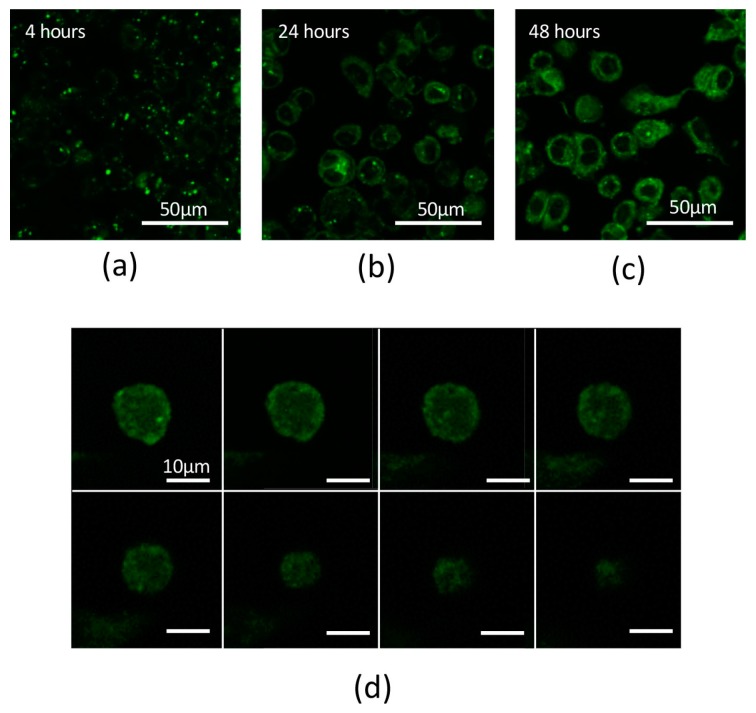
(**a**) Confocal laser scanning microscopy (LSM) image showing strong Alexa 488 fluorescence intensity on the surface of SK-BR3 cells. PCSN probes were bound to the surface of SK-BR3 cells within 4 h. (**b**) LSM image showing strong fluorescence signals inside the SKBR3 cells. PCSN probes were internalized into the cytoplasm during a 24-h incubation in medium containing targeted PCSN probes (1200 ppm). (**c**) PCSN probes remained inside the SK-BR3 cells after 48 h. (**d**) For three-dimensional analysis of the SK-BR3 cells, images were collected at 0.41-µm intervals with a 488-nm laser to create a Z stack. The images show internalized PCSN probes inside an SK-BR3 cell.

**Figure 4:**
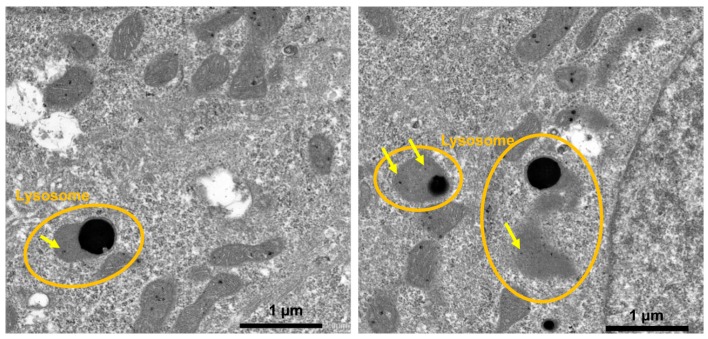
Transmission electron microscopy (TEM) images showing nanoparticles (PCSNs, yellow arrows), confirming that the probes are inside the cells. Some of the silica nanoparticles are found within lysosomes.

**Figure 5:**
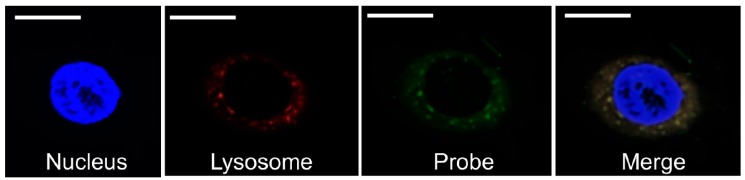
Images of a nucleus (DAPI, 4′,6-diamidino-2-phenylindole), lysosomes (Lyso Tracker Red) and probes (Alexa488). The probe and lysosomal signals overlap. Scale bars represent 10 µm.

Based on transmission electron microscopy (TEM) observations, the PCSNs utilized in this study were approximately 40 nm in size, between 35 and 46 nm (Figure 1). On thin-layer chromatography (TLC) plates, Alexa 488 fluorescent tags (Alexa Fluor^®^ 488, carboxylic acid, succinimidyl ester) did not separate from PCSN-conjugated droplets, unlike Alexa 488 applied alone as a control (Figure 2) [19], thus confirming the attachment of Alexa 488 to the PCSNs. In vitro, PCSN probes bound to the surface of SK-BR3 cells (human breast cancer cell line, HER2; 3+) within 4 h and were internalized after incubation for 24 h in cell culture medium containing dispersed PCSN probes (PCSN concentration: 1200 ppm) (Figure 3). In the previous report, stronger fluorescence signals were observed on the cell surface of SK-BR3 cells than MDA-MB231 cells as a HER2 negative control. The difference was also verified by flow cytometry analysis. These results indicate that the PCSN probes specifically target HER2 overexpressing cells [19]. For three-dimensional confocal laser scanning microscopy (LSM) analysis of PCSN probes in SK-BR3 cells, images were collected at 0.41-µm intervals with a 488-nm laser to create Z stacks. The images show PCSN probes internalized by the cells (Figure 3). TEM images confirmed that PCSNs were localized within the SK-BR3 cells and were occasionally observed inside lysosomes (Figure 4). Fluorescence images of cellular organelles revealed an overlap between lysosomes (red) and PCSN probes (green) (Figure 5).

### 2.2. Inhibition of Cell Growth by PCSNs

**Figure 6:**
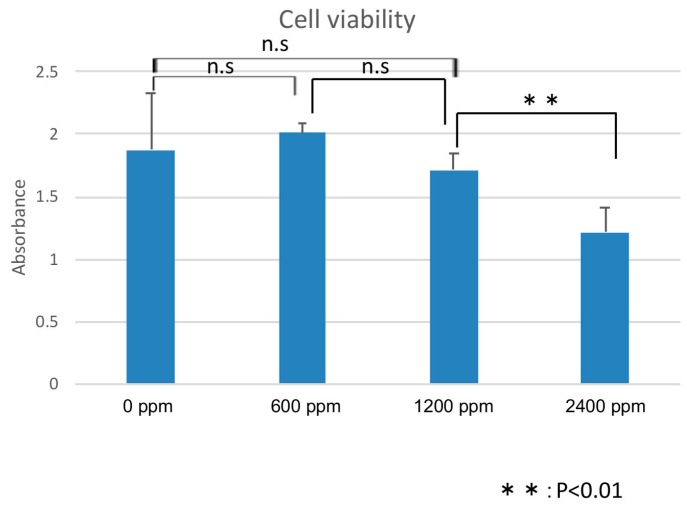
Cell viability assays show that the medium containing 2400 ppm PCSNs resulted in the lowest absorbance. The difference between 2400 ppm PCSNs and the other samples was statistically significant. Thus, a PCSN concentration of 2400 ppm damages SK-BR3 cells. The y-axis represents the absorbance unit (450 nm). n.s., not significant.

Cell proliferation assays showed no significant effects on the cell viability after 24 h of treatment with PCSNs at concentrations below 1200 ppm. PCSNs inhibited cell proliferation at 2400 ppm (Figure 6).

### 2.3. Combination of Radiation Therapy and PCSN Probes

**Figure 7:**
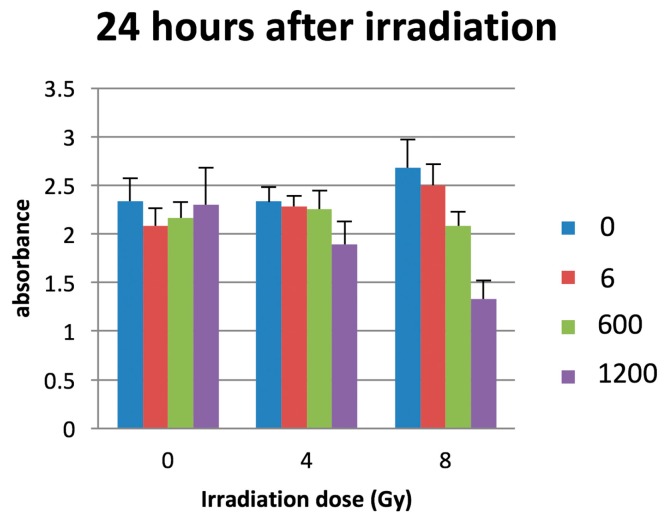
The combination of PCSN probes (PCSN concentration: 1200 ppm) and 8-Gy irradiation resulted in the lowest absorbance 24 h after irradiation. The numbers (0, 6, 600, 1200) indicate the concentration of PCSN (ppm). The *y*-axis represents the absorbance unit (450 nm).

Twenty-four hours after irradiation, cell proliferation assays showed that the combination of PCSN probes (PCSN concentrations: 6, 600, 1200 ppm) and radiation (8 Gy) decreased the survival rate of SK-BR3 cells to a greater extent than did the application of radiation alone (Figure 7). When the radiation dose was 4 Gy, there were no significant differences between samples as determined by ANOVA.

**Figure 8:**
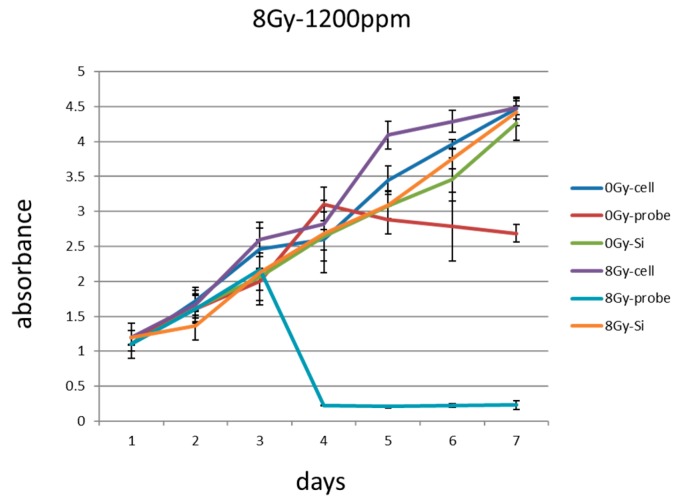
Day 1: passage the SK-BR3 cells, Day 2: add PCSN probes or silica to the cell medium, Day 3: irradiate the cells (8 Gy). In subsequent cell viability assays, the combination of 1200 ppm PCSN probes and irradiation for 24 h at 8 Gy resulted in reduced SK-BR3 cell absorbance. The *y*-axis represents the absorbance unit (450 nm). Radiation dose: 0 Gy or 8 Gy; Cell: SK-BR3 cell; Probe: containing PCSN probe; Si: containing PCSNs.

**Figure 9:**
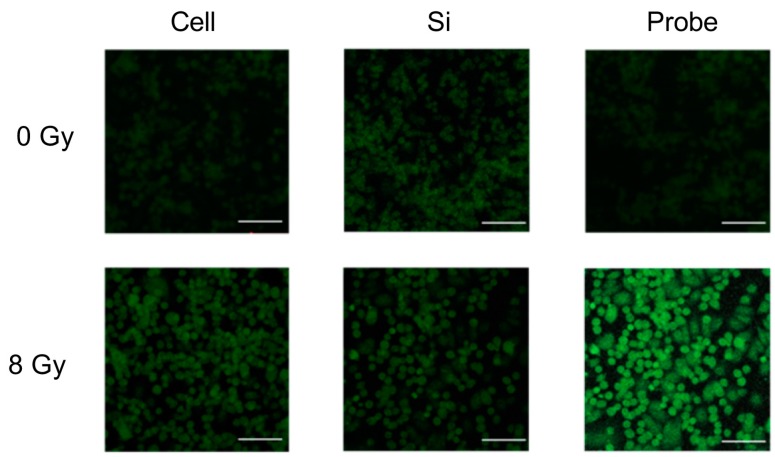
In the Terminal deoxynucleotidyl transferase (dUTP)-mediated nick end-labeling (TUNEL) assays, the three samples irradiated with 8 Gy showed stronger fluorescence intensity than the non-irradiated samples. The combination of PCSN probes and 8-Gy irradiation produced the strongest fluorescence intensity. Scale bars represent 100 µm. Cell: SK-BR3 cells alone; Si: cells in medium containing 1200 ppm PCSNs; Probe: cells in medium containing 1200 ppm PCSN probes.

**Figure 10:**
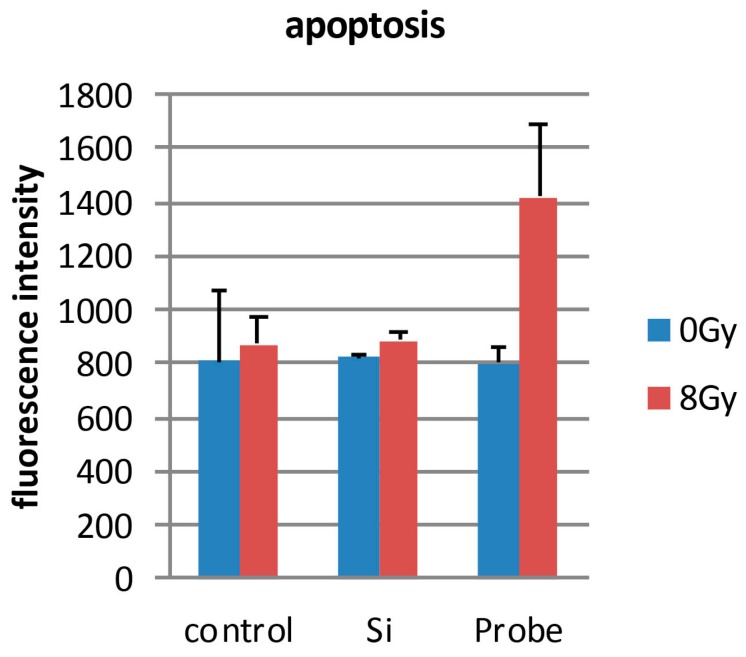
The TUNEL assay shows the highest fluorescence intensity for cells that underwent combination therapy compared to that of other samples. Combination therapy thus induced the greatest extent of apoptotic cell death. Control: SK-BR3 cells; Si: cells in medium containing 1200 ppm PCSNs; Probe: cells in medium containing 1200 ppm PCSN probes.

**Figure 11:**
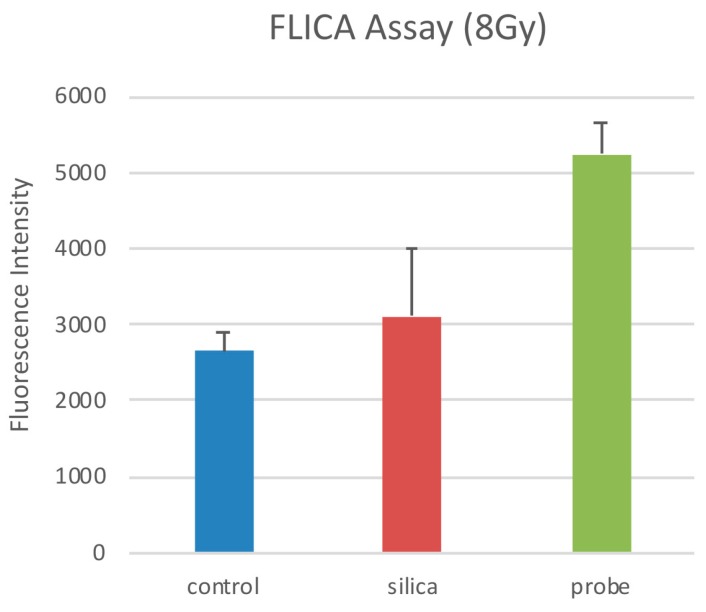
The highest fluorescence intensity resulted from the combination of PCSN probes and 8-Gy irradiation. There was a significant difference between “probe” and the other samples, showing that combination therapy caused the greatest extent of apoptotic cell death. * *p* < 0.1. Control: SK-BR3 cells, silica: cells in medium containing 1200 ppm PCSNs, probe: cells in medium containing 1200 ppm PCSN probes.

Quantifying cell viability for seven days after the cells were seeded revealed that the combination of PCSN probes and radiation (1200 ppm and 8 Gy, respectively) promoted and induced cell death (Figure 8). Terminal deoxynucleotidyl transferase (dUTP)-mediated nick end-labeling (TUNEL) assays showed that maximum apoptosis was induced by the combination of radiation and PCSN probes (Figure 9 and Figure 10). Fluorescent-labeled inhibitor of caspases (FLICA) assays showed that the combination of PCSN probes and radiation at a dose of 8 Gy resulted in the highest fluorescence intensity (Figure 11). We found that there was a lack of complete consistency of results between WST-8 and the TUNEL analysis images. This could be explained by different methodological sample preparations. However, it is apparent that the combination of the PCSN probe and the radiation induced the greatest effect on cell viability (Figure 8, Figure 9 and Figure 10).

## 3. Discussion

Although amorphous silica nanoparticle probes have previously been approved for human clinical trials, amorphous silica nanoparticles have been shown to cause cell damage [8,9]. Several previous studies found that the cell damage induced by silica nanoparticles depends on surface modifications and particle size [20,21,22]. Generally, small silica nanoparticles exert greater effects than larger nanoparticles [20,23]. However, 50-, 100- and 150-nm-sized silica nanoparticles were not shown to induce significant effects [23]. Moreover, a study comparing the cell damage caused by SiO_2_ nanoparticles revealed that there was no significant difference between 15-nm and 46-nm particles, and the cell damage they caused was dependent on the concentration and time [24]. The PCSNs used in this study were 20–50 nm in diameter and surfaced with dendrimers (Figure 1) [18]. We observed no significant difference in SK-BR3 cell viability among control cells and cells exposed to PCSNs at 600 or 1200 ppm in medium for 24 h (Figure 6). The highest tested concentration, 2400 ppm, inhibited cell growth (Figure 6). Throughout seven subsequent days of observation, PCSNs at 1200 ppm did not influence cell viability (Figure 8). PCSN probes only decreased cell viability when they were internalized by the targeted cells through a receptor-mediated endocytosis mechanism (Figure 8).

The PCSN probes bound to the surface of SK-BR3 cells within 4 h of incubation. The PCSN probes were then internalized through a receptor-mediated endocytosis mechanism within 24 h of incubation in medium, and the probes remained inside the cells for more than 48 h (Figure 3). This suggests that the PCSN probes were inside the cells during radiation treatment. Radiation most strongly influenced cell viability when it was applied in combination with PCSN probes at a high concentration (Figure 7 and Figure 8). This result suggests that the PCSN probes enhanced the effects of radiation on SK-BR cells.

How does the combination of PCSNs and radiation inhibit cell proliferation? Our histological, TEM and confocal LSM findings showed that the PCSNs were localized to lysosomes (Figure 4 and Figure 5). Furthermore, the combination of PCSN probes and radiation caused apoptosis in SK-BR3 cells (Figure 10 and Figure 11). It has been reported that silica nanoparticles induce apoptosis in several cell types [25,26,27,28]. Partial permeabilization of lysosomal membranes induces apoptosis, whereas more massive lysosomal ruptures induce necrosis [29,30,31]. Internalized silica nanoparticles may disturb the permeability of the lysosomal membrane, and the resulting lysosomal destabilization leads to cell destruction and autophagy [32,33,34].

The major effect of ionizing radiation on cells is direct damage to the DNA, which occurs through two mechanisms: direct breakage of DNA strands and indirect breakage by free radicals, which are largely produced by the radiolysis of water. It has been reported that an additional target of ionizing radiation is the lysosome, in which iron-dependent reactive oxygen species are produced to promote lysosomal membrane permeabilization, thereby activating cell death [35,36,37].

**Figure 12:**
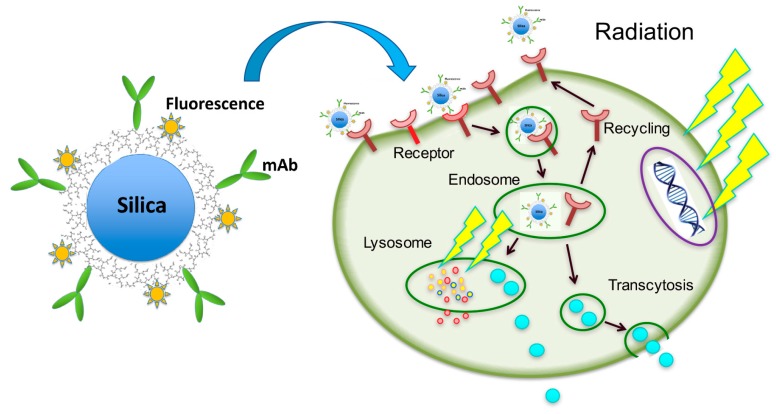
Possible mechanism for targeted radiosensitization by PCSN probes. Internalized silica nanoparticles may disturb the permeability of the lysosomal membrane in the targeted cell (HER2 positive), and the resulting lysosomal destabilization leads to cell destruction and autophagy. The major effect of ionizing radiation on the cell is direct damage to the DNA, and possible additional effect can also damage lysosomal membrane permeabilization, thereby activating cell death. The combination of PCSN probes and radiation may thus cause cell growth inhibition and cell death through lysosomal membrane destabilization. Anti-HER2 antibody on PCSN probes can also inhibit the MAPK and PI3K pathways, leading to the suppression of cell growth and proliferation. Yellow arrow signals: ionized radiation; mAb: Anti-HER2 antibody; Orange star-like dots around silica nanoparticle: fluorophore; Blue dots in the cell: degraded silica nanoparticle.

The combination of PCSN probes and radiation may thus cause cell growth inhibition and cell death through lysosomal membrane destabilization. Furthermore, Trastuzumab, a humanized monoclonal antibody, selectively binds to the extracellular domain of the HER2 receptor. Trastuzumab triggers HER2 internalization and degradation by promoting tyrosine kinase activity [38] and inhibits the MAPK and PI3K pathways, leading to the suppression of cell growth and proliferation. We used anti-HER2 antibody (HER2/ErbB2 (D8F12) XP Rabbit mAb) for PCSN probes that also binds to the extracellular domain of the HER2 receptor as Trastsuzumab (Herceptin) does. PCSN probes may block these pathways because the probes are surfaced with monoclonal antibodies to target HER2. It has been reported that trastuzumab, when combined with dendrimers, traffics to and localizes within lysosomes [39,40]. Thus, we hypothesize that the observed reduction in cell proliferation is largely related to lysosome dysfunction (Figure 12). However, this hypothesis requires further study.

The approach described in this study increases the efficiency of radiation treatment and greatly enhances its growth inhibition effects against HER2-overexpressing cancer cells. This suggests that PCSN probes act as radiosensitizers, leading to higher efficacy of radiotherapy against HER2-overexpressing cells without harming normal cells. Moreover, PCSN probes may have a dual imaging and radiosensitizing functionality against their specific target cells, as suggested by previous reports using PAMAM silica nanoparticles as dual imaging probes [18,19]. This suggests that PCSN probes may also be useful as theranostic probes.

## 4. Materials and Methods

### 4.1. PCSN Probes

**Figure 13:**
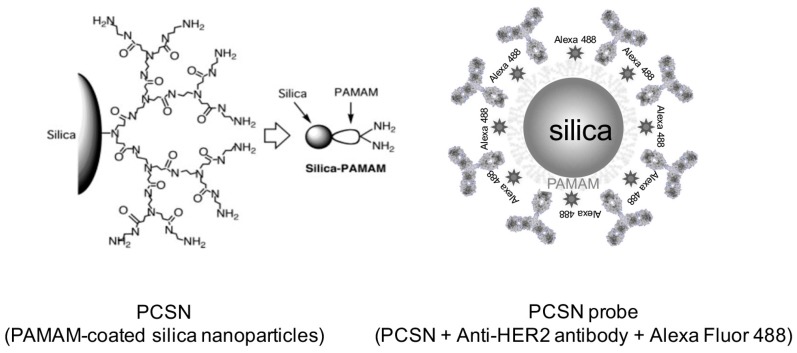
PCSN: Hyperbranched polyamidoamine (PAMAM) is grafted onto the surface of an amorphous silica nanoparticle to functionalize the particle. PCSN probe: PCSN is conjugated with a fluorescent dye, Alexa Fluor 488, and anti-HER2 antibodies are covalently attached to the labeled PCSN.

Hyper-branched PAMAM (generation 3) was grafted onto the surface of synthetic amorphous silica nanoparticles (Aerosil 200, Nippon Aerosil, Tokyo, Japan) (Figure 13), resulting in a particle size of 20–50 nm, as previously reported [6]. The percentage of grafted PAMAM was 55.6%. Forty milligrams of the PCSNs were dissolved in 40 mL of 50% ethanol/distilled deionized water, mixed using an ultrasonic water bath for 30 min and centrifuged (11,000 rpm for 5 min) in spin columns (Ultrafree centrifugal filter, Durapore PVDF 0.1 µm, Millipore, Billerica, MA, USA) for purification. The concentration of PCSNs in the purified solution was 156.69 mg/L. PCSNs were then conjugated to a fluorescent dye at 37 °C for 30 min. The fluorescent dye was composed of Alexa 488 (Alexa Fluor^®^ 488, carboxylic acid, succinimidyl ester, Thermo Fisher Scientific, Waltham, MA, USA) dissolved in 1 mL of dimethyl sulfoxide (DMSO, Thermo Scientific, Hudson, NH, USA). The Alexa 488 solution was then diluted 50-fold with PCSNs. The conjugation of Alexa 488 to the PCSNs was verified with TLC, utilizing TLC silica gel 60 F254 (Merck KGaA, Darmstadt, Germany), a 50% ethanol solution and a phosphor imager (FLA-2000, FUJIFILM, Tokyo, Japan). Ten microliters of anti-HER2 antibodies (HER2/ErbB2 (D8F12) XP Rabbit mAb, Cell Signaling Technology, Danvers, MA, USA) and 0.8 mg of a coupling reagent, 1-ethyl-3-(3-dimethylaminopropyl) carbodiimide hydrochloride, dissolved in 80 μL of reaction buffer (following the manufacturer’s instructions; Peptide Coating Kit, TaKaRa, Shiga, Japan) were added to 200 µL of fluorescently labeled PCSNs and incubated for 30 min at 37 °C. The carbodiimide reaction was then used to couple PAMAM amines with the carboxyl-terminal regions of antibodies utilizing the kit. The 100 µL of prepared PCSN probe solution was mixed with 200 µL PBS and stirred for 5 min. The mixture was centrifuged (11,000 rpm for 5 min). Two hundred µL of supernatant was removed and then 200 µL of PBS was added before another centrifugation for purification. Two hundred µL of supernatant was removed again and the residue was used for the experiment. The silica concentration of the solution was 0.6 mg/L.

### 4.2. Cell Culture

The human breast cancer cell line SK-BR3 (HER2; 3+) [7] was obtained from the American Type Culture Collection (ATCC^®^ number HTB-30TM). The SK-BR3 cells were cultured in McCoy’s 5A medium (GIBCO^®^, Thermo Fisher Scientific, MA, USA) supplemented with 10% fetal bovine serum (FBS, GIBCO^®^, Thermo Fisher Scientific, MA, USA) and 1% penicillin-streptomycin (Thermo Fisher Scientific, MA, USA). The cell line was maintained in a humidified environment containing 5% CO_2_ at 37 °C. The medium was changed every other day.

### 4.3. Internalization of PCSN Probes by HER2-Overexpressing Cells

For each experiment, 2 × 10^5^ SK-BR3 cells were seeded into each well of a 24-well plate. After 24 h, PCSN probes (PCSN concentration: 1200 ppm) were added to the medium and then incubated for 4 h, 24 h or 48 h. After washing with phosphate buffered saline (PBS), the samples were examined by confocal LSM (LSM710, ZEISS, Oberkochen, Germany) with a fluorescent filter (fs38HE: Ex. 470/40 nm).

### 4.4. Transmission Electron Microscopy (TEM)

In total, 2 × 10^5^ SK-BR3 cells were seeded onto individual plastic cover slips. After 24 h, PCSN probes were added and incubated for 24 h. Slides were then placed in a 4% paraformaldehyde-0.2% glutaraldehyde fixative in 0.1 M phosphate buffer (PB, pH 7.4) for 2 h at room temperature and were rinsed with 0.1 M PBS for 5 min at room temperature three times. After rinsing, the coverslips were cut into small pieces (3 × 3 mm), post-fixed for 1 h in 1% osmium tetroxide at room temperature, and then quickly rinsed twice in distilled water. The coverslips were embedded in epoxy resin after dehydration through a graded alcohol series. Semi-thin sections were cut, stained with toluidine blue and examined under a light microscope. Ultrathin sections were cut with a diamond knife (Diatome, Biel, Switzerland) on an ultramicrotome (Reichert Jung, Vienna, Austria) and stained with platinum blue (TI blue, Nisshin EM Co, Tokyo, Japan)/uranyl acetate and lead citrate. The sections were examined using TEM (JEM-1010, JEOL, Tokyo, Japan).

### 4.5. Evaluation of Cell Viability Using Several Concentrations of PAMAM Silica Nanoparticles

In total, 3 × 10^5^ SK-BR3 cells were seeded into each well of a 96-well plate. After 24 h, PCSNs, at a concentration of 0, 600, 1200 or 2400 ppm, were added to the medium, and the cells were incubated for 24 h. After the cells were washed with PBS, they were analyzed using Cell Counting Kit-8 (Dojindo Molecular Technologies, Kumamoto, Japan) assays according to the manufacturer’s protocol. Cell Counting Kit-8 is a convenient assay using WST-8 (2-(2-methoxy-4-nitrophenyl)-3-(4-nitrophenyl)-5-(2,4-disulfophenyl)-2*H*-tetrazolium, monosodium salt), which is reduced by cellular dehydrogenases to produce an orange formazan product. The amount of formazan produced is directly proportional to the number of living cells, which means that this assay facilitates the analysis of cell viability via the direct measurement of formazan absorbance at 450 nm (Viento 808, DS PHATMA BIOMEDICAL, Osaka, Japan).

### 4.6. The Combination of PCSN Probes and Irradiation

A total of 3 × 10^5^ SK-BR3 cells were seeded per well in a 96-well plate. After 24 h, PCSN probes at a concentration of 0, 600 or 1200 ppm were added to the medium. The cells were incubated for 24 h. After the medium was refreshed, the cells were exposed to X-rays with graded doses (0, 4, 8 Gy) radiated from a medical linear accelerator (Primus Mid-Energy Single Photon type M2-6300 (4MV X-ray, 250cGy/min, TOSHIBA Medical Systems, Tochigi, Japan).

### 4.7. Observation of Cell Viability

The cell viability of the six samples, defined as the absorbance from Cell Counting Kit-8 assays, was followed for seven days after seeding. The following schedule was used: Day 1, pass the SK-BR3 cells; Day 2: add PCSN probe or silica to the cell medium; Day 3: irradiation (8 Gy). The medium was changed every other day.

### 4.8. TUNEL Assay

SK-BR3 cells were seeded on coverslips at the bottom of the wells in a 24-well plate and exposed to PCSN probes (1200 ppm) and/or X-rays (8 Gy). Twenty-four hours after irradiation, TUNEL staining was performed to detect apoptosis-specific nuclear DNA fragmentation. The cells were observed by fluorescence microscopy (Axiovert 25, ZEISS, Oberkochen, Germany), and fluorescence intensity was measured using a microplate reader (Power Scan’MX, DS PHARMA BIOMEDICAL, Osaka, Japan).

### 4.9. FLICA Assay

Cells were cultured in 96-well plates and treated with probes at a silica concentration of 1200 ppm for 24 h. After refreshing the medium, the cells were exposed to X-rays (8 Gy). FLICA was used to detect apoptotic cell death according to the manufacturer’s guidelines. The FLICA methodology is based on the use of a non-cytotoxic and cell-permeable fluorochrome inhibitor of caspases, which binds covalently to active caspases and emits green fluorescence. Apoptotic cell death was quantified using a fluorescent plate reader (Power Scan’MX, DS PHARMA BIOMEDICAL, Osaka, Japan).

### 4.10. Fluorescent Staining of Cell Organelles

Cells were seeded on coverslips at the bottom of the wells in a 24-well plate and were treated with Alexa 488-PCSN probes or PCSN and/or X-rays. After removing the culture medium from each well, lysosomes were dyed with LysoTracker Red (Thermo Fisher Scientific), and nuclei were dyed with 4′,6-diamidino-2-phenylindole (DAPI) (Antifade Mounting Medium with DAPI, VECTASHIELD, Vector laboratories, Burlingame, CA, USA) according to the manufacturer’s product data sheet. Subsequently, the coverslips were observed by confocal LSM (LSM710, ZEISS, Oberkochen, Germany) with filters set for DAPI (Ex/Em: 350/470 nm), Texas Red (Ex/Em: 540/605 nm) and GFP/FITC (Ex/Em: 488/514 nm).

### 4.11. Data Analysis

All statistical analysis of cell viability and the number of apoptotic cells was performed with ANOVA.

All animals were treated in accordance with the Ethical Guidelines for Investigations of Experimental Animals of the Nippon Dental University School of Life Dentistry at Niigata (No. NDU-N-2015-123, 19 May 2015).

## 5. Conclusions

The results of this study demonstrated that HER2-targeted PCSN probes act as specific radiosensitizing agents in SK-BR3 cells. This approach is useful to image specific cells and as a targeted therapy to decrease the side effects of radiotherapy in cancer patients. Additional tumor xenograft animal studies will be required to verify whether this radiosensitization is effective in promoting tumor regression.
